# Temporal and spatial variations in the frequency of compound hot, dry, and windy events in the central United States

**DOI:** 10.1038/s41598-020-72624-0

**Published:** 2020-09-24

**Authors:** Ameneh Tavakol, Vahid Rahmani, John Harrington

**Affiliations:** 1 Department of Biological and Agricultural Engineering, Kansas State University, 1016 Seaton Hall, Manhattan, KS 66506 USA; 2 Department of Geography, Kansas State University, 1002 Seaton Hall, Manhattan, KS 66506 USA

**Keywords:** Climate change, Natural hazards

## Abstract

Simultaneous low humidity, high temperature, and high wind speeds disturb the water balance in plants, intensify evapotranspiration, and can ultimately lead to crop damage. In addition, these events have been linked to flash droughts and can play a critical role in the spread of human ignited wildfires. The spatial patterns and temporal changes of hot, dry, and windy events (HDWs) for two time periods, 1949 to 2018 (70-years) and 1969 to 2018 (50-years) were analyzed in the central United States. The highest frequencies of HDWs were observed at stations in western Kansas and west Texas. Annually, the highest number of events happened concurrently with the major heat waves and droughts in 1980 and 2011. Temporally, an overall decrease in the HDWs was significant in the eastern regions of North Dakota and South Dakota, and an upward trend was significant in Texas and the western part of the Great Plains. Significant trends in HDWs co-occurred more frequently with significant trends in extreme temperatures compared with low humidity or strong wind events. The results of this study provide valuable information on the location of places where HDWs are more likely to occur. The information provided could be used to improve water management strategies.

## Introduction

The World Meteorological Organization (WMO) has defined climate extremes as infrequent climatological and meteorological phenomena that surpass a stated threshold^1^. An increase in the probability of extreme weather events was reported by the Intergovernmental Panel on Climate Change (IPCC)^2^ that corresponds with the rise in the average number of annual disasters in the United States from 10 to 35 in less than 50 years^3^. Extreme events influence the environment and society resulting in the loss of habitat, property, and even life^4^. Negative impacts from extreme events are more significant when events happen simultaneously^5^. Compound extreme events are (1) two or more extreme events occurring successively or simultaneously, (2) combinations of extreme events that amplify the impact of the events on underlying conditions, or (3) combinations of events that are not extremes by themselves but lead to an extreme event when combined^6^.

A hot, dry, and windy event (HDW)^7^ is a compound extreme that merits further examination. While any single extreme of either high temperature, low humidity, or high wind speed could have negative influences, the combination of these weather elements can have a significant impact on crop yield^8–13^, flash drought^14,15^, and wildfires^16,17^. Generally, plant water demands for growth and evaporative cooling of tissues increase on sunny days with dry winds^18^. As the soil dries due to evaporation and plant transpiration, the soil water below the surface starts moving upward by capillary action. If evapotranspiration continues and the near surface soil becomes drier, resistance in the rate of water flow in the soil can become limited^18^. Limiting soil evaporation changes the partitioning of solar energy (from latent to sensible heat), with a warming of the soil surface and the air above the soil^18^. Transport of energy between the vegetative cover and the atmosphere depends on the nature of the plants, their immediate environment, and the physical characteristics of the atmosphere^19^. The amount of energy transferred by advection (horizontal transfer of a substance) depends on air temperature, humidity, and wind speed^18^. In regions with a higher frequency of HDWs, advection can provide as much energy as net radiation to drive evapotranspiration^18^. When the rate of the moisture transpired from the leaves is faster than the rate of moisture received from the roots, plant damage can begin.The amount of damage depends on the length of time plants are exposed to these conditions^10^. In more humid regions with lower wind speeds, the contribution of advection to the rate of evapotranspiration is considerably less. Overall, HDWs can affect crop yield and cause significant economic impact. Lydolph^10^ explained the correlation between the distribution of HDWs and crop yields, with a higher destructive impact occurring in unirrigated croplands. During a HDW event it is hard to accurately calculate evapotranspiration^20^ and the latent heat flux^21^.

Although precipitation deficit is the basic requirement for flash drought, HDWs influence on the development speed and severity of droughts^14,15^. In addition to the impact of HDWs on croplands and drought, other studies have documented the critical role of HDWs in the spread of wildfires in Europe^22^, Mediterranean region^23^, and United States^16,17^. One study^24^ examined the climatology of an atmospheric index that combines stability with dryness to estimate erratic wildfire behavior in the United States. Maps from the climatic analysis document areas of high risk for the southern Great Plains. These areas can have extended periods of consecutive days of high risk. The occurrence of HDW compound events may lead to more severe wildfires^25,26^. Temperature, relative humidity, precipitation, and wind speed are weather variables that influence the intensity and speed of wildfires. An ability to forecast these variables has been used to develop a fire-weather prediction index to predict when the weather may make wildfires more difficult to manage^27^. McDonald et al.^28^ described the climatology of HDW index for the contiguous United States using data from the Climate Forecast System Reanalysis. Days with HDW values higher than the 95th percentile lead to the most dangerous fire behaviors.

An increase in global temperature^2,29,30^ is expected to substantially increase the probability of compound extremes^31–33^. High temperature, low humidity, and high-speed winds are the components of HDWs. The annual average temperature has increased in the United States since 1895^2,34^. However, the mean annual^35,36^ and mean monthly wind speed^37^ have declined. The change in relative humidity was not significant globally^2^. In the United States, the trend of relative humidity is weak with the maximum increase in winter^38^. Considering the shifts in temperature, wind speed, and relative humidity, the aim of this study is to better understand the spatial patterns and temporal variation in compound HDWs in the central United States. First, this study gathered existing definitions of HDWs from peer-reviewed studies. Then, the spatial and temporal changes of HDWs were analyzed in the central United States applying two different periods. HDWs between the two periods were compared to analyze whether the length of data and the number of stations would result in different spatial and temporal changes in HDWs.

## Background

Desiccated corn acreage in southern Kansas in 1888^39^ provides a historic example of the impact of HDWs on cropland. An extended and severe drought was reported in Kansas in 1887 and in the next year (1888) a series of hot winds destroyed 30% of the corn crop in south-central Kansas. Between 1883 and 1888 nearly 40 occurrences of HDWs were reported in the central United States including locations in Kansas, Nebraska, the Dakotas, Texas, and Arkansas^39^. The majority of the events were in June, July, and August. In September 1931 and following a month without rain, seven consecutive days of hot winds were reported at Ashland, Kansas, including a report of 49 °C (120 °F) for September 5th^40^. Similar reports from observers in the Great Plains and other states documented crop damage from only a few hours of HDWs.

A variety of terms and definitions are used in the literature to describe HWDs. Lydolph and Williams^11^ suggested considering the co-occurrence of low relative humidity, high temperature, and stronger winds to define an hourly HDW event. They identified five different classes of HDWs based on relative humidity less than 30%, wind speed less than or greater than 7 m/s, and temperature higher than 29 °C (Table 1). Leathers and Harrington^9^ classified HDWs or “furnace winds” with a temperature higher than 35 °C (a threshold considered critical for crop development), relative humidity lower than 30%, and wind speed equal to or greater than 7 m/s. Reid et al.^17^ categorized wildfires in Oklahoma into six classes and showed that different classes of wildfires are more probable when the average wind speed is in the range of 3.5 m/s (for class 1) to 7 m/s (for class 6) when relative humidity ranges from 15 to 30%. Kruger et al.^16^ found that in the southern Great Plains, the favorable condition for wildfire in the growing season includes a median relative humidity of 18–30%, the temperature of 34–41 °C, and wind speed of 6.5–8.6 m/s.

**Table 1:** Definition and categorization of hot, dry, and windy events (HDWs) based on different studies.

Classification	Temperature (°C)	Relative humidity (%)	Wind speed (m/s)	References
1	> 29	< 30	–	Lydolph and Williams^11^
2	29–37	< 30	< 7	
3	29–37	< 30	≥ 7	
4	≥ 38	< 30	< 7	
5	≥ 38	< 30	≥ 7	
–	> 35	< 30	≥ 7	Leathers and Harrington^9^
Slight	> 32	< 30	> 2	Wang et al.^12^
Severe	> 35	< 25	> 3	
–	> 30	< 30	> 2	Huailiang et al.^51^ and Liu and Kang^52^
Light	≥ 32	≤ 25	≥ 3	Shi et al.^53^
Heavy	≥ 35	≤ 30	≥ 3	

Temperature changes influence crops depending on the crop type. For instance, while the observed increase in temperature during 1968–2013 was beneficial for maize yields in the Great Plains, it showed a detrimental effect on soybean and sorghum yields^41^. A negative impact of temperatures higher than 34 °C on different crops has been documented in the United States^42^. Table 1 shows several definitions and classifications of HDWs. In this study, hourly data observations were used to count the number of HDW events.

For the impact of wind speed, a 25% reduction in grass leaf extension was discovered with an increase of wind speed from 1 to 7.4 m/s^43^. Besides, a 50% increase in Helianthus annuus (sunflower) water requirement^44^ and a 50% reduction in the dry weight of marigolds grown^45^ when wind speed is higher than 7 m/s (15 miles per hour).

Our analysis indicates that in the Great Plains, the average wind speed is 4.5 m/s and 4.3 m/s for 70-years and 50-years periods, respectively. Wind speed of 7 m/s is equal to the 85th percentile threshold for both periods. The 90th percentile of wind speed for the entire Great Plains is equal to 8.2 m/s and 7.7 m/s for 70-years and 50-years periods, respectively. Considering the mentioned information in the literature and high wind speed in the Great Plains, the 7 m/s threshold was considered which is also the same as in previous studies^9,11^ that analyzed the geography of HDWs in the United States.

For relative humidity, a leaf water deficit and small root water deficit were reported for relative humidity values between 30 and 35%^46^ or about 25%^47^. Hoffman et al.^48^ analyzed the impact of low (25%) and high (90%) relative humidity on cotton plants and found a significantly higher leaf diffusion resistance when the humidity is low. O’Leary and Knecht^49^ revealed that the yield of bean plants decreased by 40% when the RH decreased from 70–75 to 35–40%. For Lettuce sativa, growth will decrease when the relative humidity decreases to 35–40%^50^.

Seasonally, a majority of HDWs are reported in late spring and summer worldwide. In Siberia, the “Sukhovey” winds occur primarily in May and June, with very few in late summer^10^. In the Great Plains, USA, HDWs mainly occured in mid-summer, however, the less-frequent HDWs of the southeastern United States occur primarily during less humid periods in September, June, or May^11^. In China, most HDWs are reported in May and June^12^. A study from a wind tower in Algeria documented a HDW event blowing from the deserts during the hot season with a wind speed of 4.7 m/s in July^54^. Across Africa, the “Khamsin”, “Sharav”, and “Sirocco’’ HDWs mostly occur in late spring and early summer. As HDWs are usually expected between May and September, the warm season in the central United States (May through September) was selected for this study.

## Results and discussion

**Figure 1:**
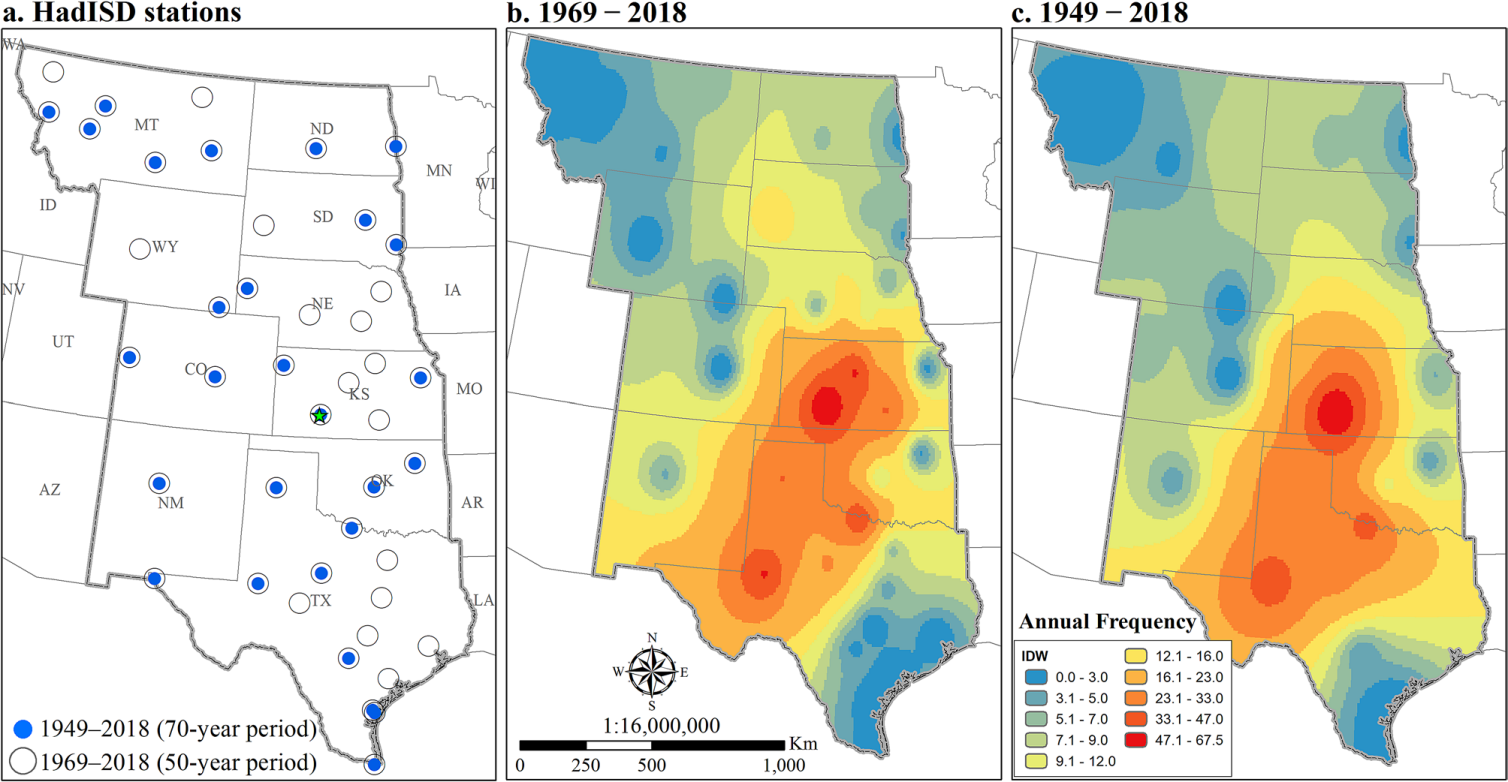
Location of stations analyzed in the central United States. The number of available stations increased from 27 to 44 when the study period decreased from 70 to 50 years (**a**). Annual mean frequency of hourly warm-season HDWs in 50-years (**b**) and 70-years (**c**) periods using Inverse Distance Weighted (IDW) interpolation method. The highest values were determined for Dodge City, Kansas, for both periods. The figure has been generated with ESRI-ArcGIS, version 10.6 (https://www.esri.com/arcgis/about-arcgis).

Two periods were selected to study temporal and spatial changes in HDWs. A 70-years period (1949–2018) was studied using 27 stations and a 50-years period (1969–2018) was assessed using 44 stations (Fig. 1). The frequency of hourly HDW events was determined at each station for each month in the warm season. Then, monthly values were summed to calculate an annual (warm season) total. No matter which time period was considered, the highest annual frequencies of HDWs occurred at stations in western Kansas and Texas (Fig. 1b,c). Across all stations analyzed, the mean annual frequency of HDWs ranged from less than 1.0 to more than 60.0 h, with the largest number occurring in southwest Kansas (Dodge City). This finding reinforces results from a previous study analyzing 1948–1993 data that identified Dodge City, Kansas, as the hotspot^9^. This location coincides with a high-speed-wind region in the United States^55–58^. Analysis of the geographic pattern of mean monthly wind speed for 1961–1990 showed the highest values in the Great Plains, with the largest values in Kansas and Texas in June, July, August, and September^55^. Spatial analysis of wind-power density showed the highest classes of wind speed (greater than 7 m/s) in southwest Kansas, northwest Nebraska, and North Texas in the summer^58^. Using the information in the 2011 National Land Cover Database (NLCD^59^), greater frequencies of HWDs were located in croplands and grasslands.

Four interpolation methods including Inverse Distance Weighted (IDW), Natural Neighbor, Ordinary Kriging, and Universal Kriging were used to interpolate the point-based data. Two statistical error indicators including the mean absolute error (MAE) and root mean square error (RMSE) were then used to test the results of the interpolation methods. IDW was then selected based on the minimum error calculated for both MAE and RMSE.

**Figure 2:**
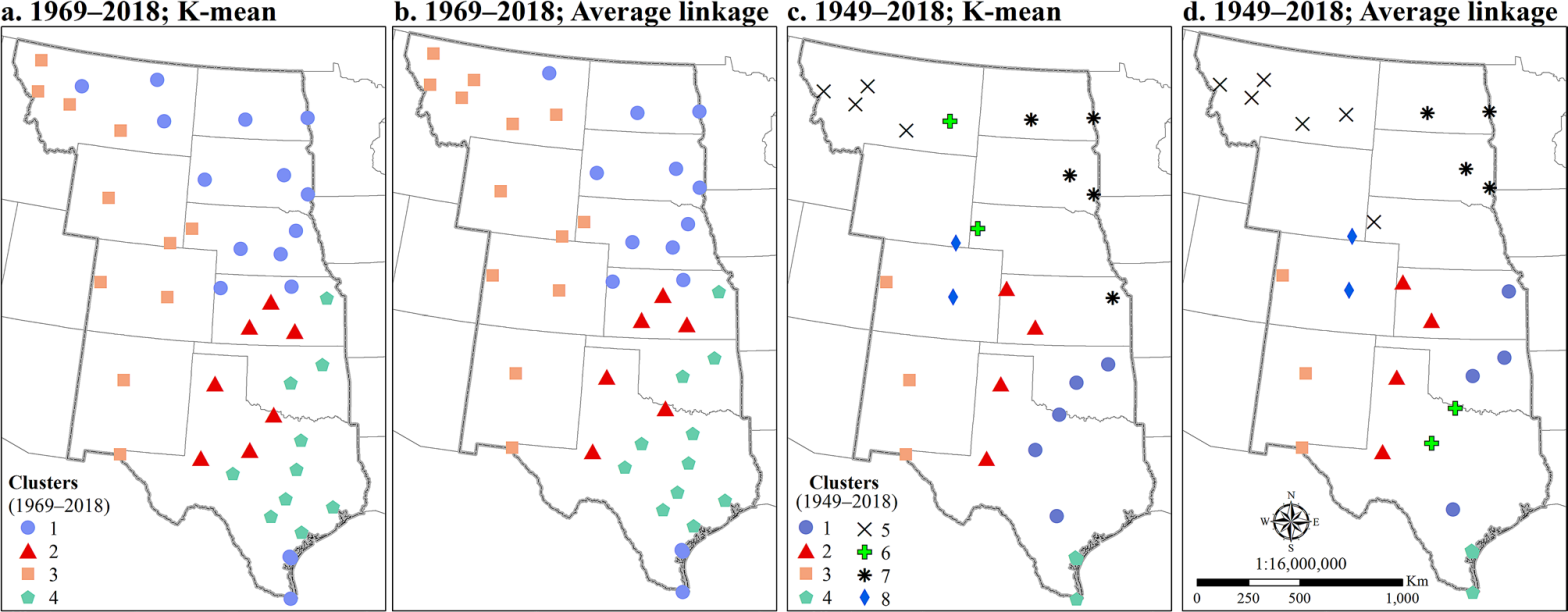
Grouping the stations in the central United States based on the frequency of single and compound extreme events for the 50-years (**a**,**b**) and 70-years (**c**,**d**) periods using K-mean and average linkage methods. Clusters were created by R software, factoextra-package Version 1.0.7, https://CRAN.R-project.org/package=factoextra and cluster-package Version 2.1.0, https://CRAN.R-project.org/package=cluster). The final maps in this figure have been generated with ESRI-ArcGIS, version 10.6 (https://www.esri.com/arcgis/about-arcgis).

Then, average linkage^60^ and K-mean^61,62^ clustering methods were applied to cluster the data based on the frequency of HDWs, extreme high temperature (higher than 35 °C), extreme low relative humidity (less than 30%), and extreme high wind speed (higher than or equal to 7 m/s). First, the optimal number of clusters were specified using silhouette method^63^ and then the clusters were visualized as maps (Fig. 2). Based on the silhouette method^63^, four and eight clusters were specified as the optimal number of clusters for the 50-year and 70-year periods, respectively. The clustering results were a little different based on the two methods. However, the main grouping pattern were the same especially for the 50-year period (Fig. 2a,b). Cluster 1 groups together stations with lower numbers of HDW events and proportionally high number of extreme high wind speed events that includes stations along the Texas coast and those in the northeastern part of the study area. Cluster 2 includes the stations from central Kansas south and westward into the Texas Panhandle. These are the stations that are most at risk of having a HDW event. Cluster 3 groups stations with the highest number of extreme low relative humidity events and includes stations in the west. From eastern Kansas southward into east Texas, stations are grouped as cluster 4 (Fig. 2a,b). These stations recorded a higher number of extreme high temperature but a lower number of extreme low relative humidity and extreme high wind speed events. Clustering of the longer time series, produces a larger number of optimal clusters, with Cluster 2 representing the stations with the highest number of HDW events (Fig. 2c,d). The additional clusters produced with the analysis of 70 years of data suggest within group differences for the 50 year solution. For example, the low frequency of HDW events cluster from the 50-year analysis (Cluster 1) include Clusters 4 and 7 in the 70-year analysis (Fig. 2).

**Figure 3:**
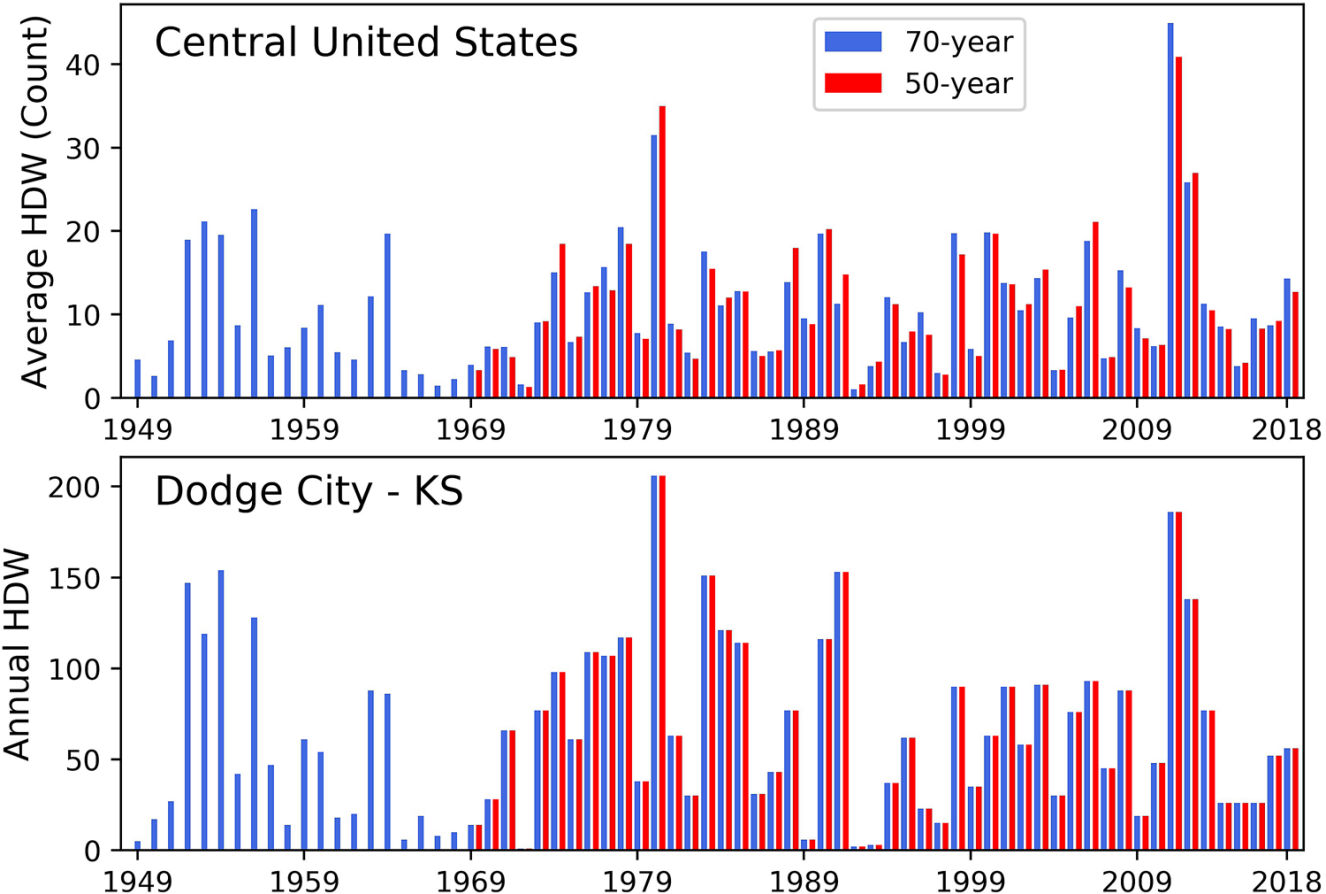
The annual multi-station average frequency of hourly HDWs in 50-years (red) and 70-years (blue) periods for all stations in the Great Plains (top) and for Dodge City (bottom). The highest frequencies occurred in 2011 and 1980. Dodge City, in southwest Kansas, recorded the highest frequency of HDWs among all stations. The difference between 50-years and 70-years average frequency values for the central United States is due to the different number of stations analyzed for each period.

Year-to-year variation in the occurrence of HDWs in the central United States was determined based on calculating the average occurrence of HDWs across all stations. Temporally, the highest average number of HDWs in the central United States occured during the droughts of 2011, 1980, and 2012 (Fig. 3). For the 70-years period, the highest averages of annual HDWs were 45 and 31 in 2011 and 1980, respectively. For the 50-years period, the average frequency of HDWs was 41 and 35 in 2011 and 1980, respectively (Fig. 3). In the summer of 1980, a major concurrent drought and heatwave caused severe agricultural damage and about 10,000 excess human deaths from the direct and indirect influences of heat stress^4^. The summer was unusually dry and hot, especially in the southern and central Great Plains, with an estimated $16 billion in economic losses^64^. The hot and dry conditions started in June and continued until late November^4^.

The major heat wave and drought of 2011 began in March and continued until late August. The concurrent drought and heat wave of 2011 caused about $13.9 billion in economic losses with major impacts in Kansas, Texas, Oklahoma, New Mexico, and Arizona^4^. In the summer of 2011, Texas and Oklahoma recorded their hottest summer, based on long-term records dating back to 1895^65^. In the three consecutive years of drought (2010–2012), 2011 was the most severe whole growing season drought in Kansas, Texas, and Oklahoma that affected the majority of tallgrass prairie regions^66^. Prolonged periods of HDWs in May and June 2012 caused a rapid deterioration of soil moisture and crop conditions and intensified the drought^67^.

**Figure 4:**
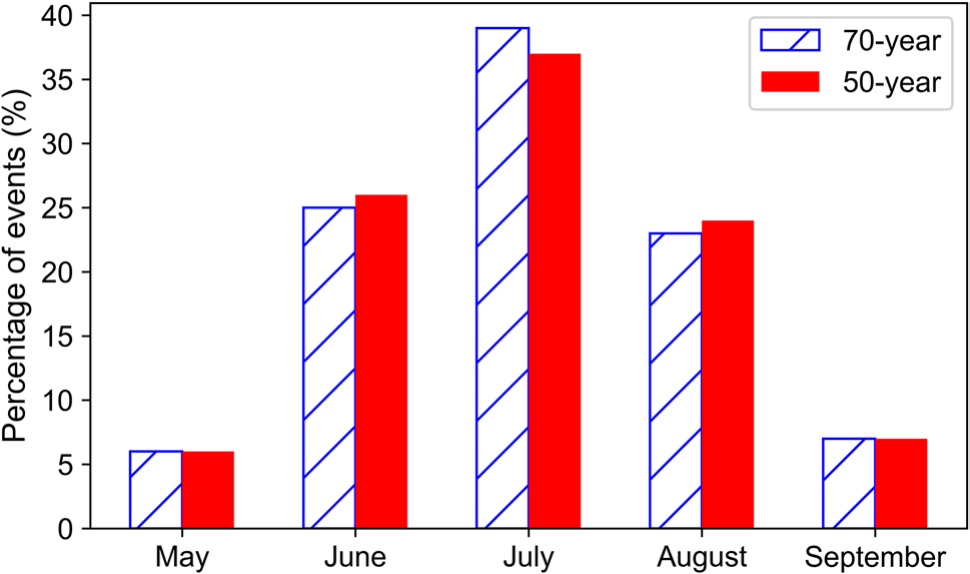
The percentage occurrence of events (HDWs) in different months for the 50-years (gray) and 70-years (black) periods. The percentages were calculated based on all events that occurred in the 50-years and 70-years periods in the central United States.

The monthly frequency of hourly HDWs showed the same pattern of occurrence for both periods with a maximum in July (Fig. 4). Analyzing the monthly pattern of single variable extremes showed the highest probability of extreme temperature (higher than 35 °C) occurred in July and August. However, extreme wind events (higher than or equal to 7 m/s) occurred mostly in May and June and the distribution was almost equal in all months for dry relative humidity extremes (less than 30%).

**Figure 5:**
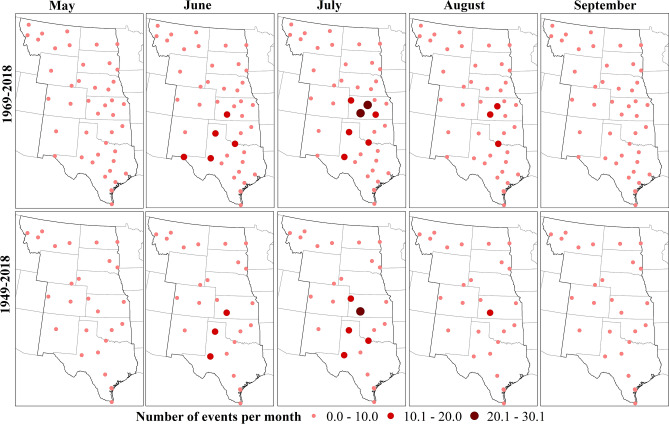
Spatial pattern of monthly frequency (average number of hourly events) of HDWs for 50-years (top) and 70-years (bottom) periods. Highest mean values were in July, June, and August, respectively. Stations in Kansas and Texas had the highest frequencies in summer months. The figure has been generated with ESRI-ArcGIS, version 10.6 (https://www.esri.com/arcgis/about-arcgis).

Stations in the southern Great Plains had the highest frequencies of monthly values of HDWs (Fig. 5). Extreme temperature events mostly occurred in Texas, Kansas, and Oklahoma. For relative humidity, the majority of extreme events took place in the western Great Plains. For wind, Texas and Kansas had the highest frequency of wind events greater than or equal to 7 m/s. Correlation analysis, based on the Pearson and Spearman method, showed no significant relationship between the annual frequency of HDWs and the annual frequency of extreme relative humidity. However, a significant correlation was determined for both wind and temperature extremes with HDWs.

**Figure 6:**
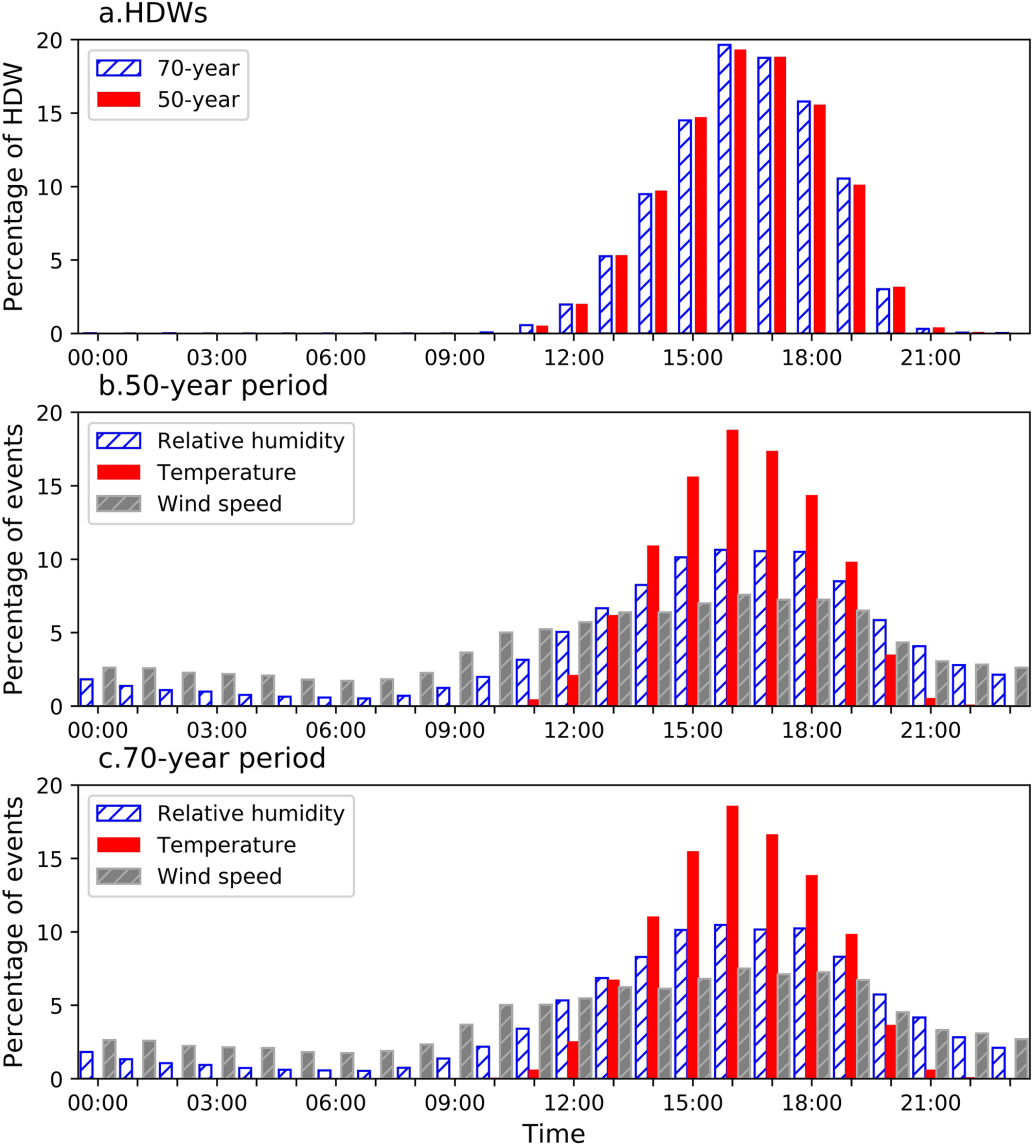
Diurnal pattern of hourly HDW occurrences in 50-years (red) and 70-years (blue) periods for all stations in the Great Plains. The greatest percentage of compound HDWs occurred in the afternoon at 4:00 and 5:00 p.m. A similar temporal pattern was determined for Dodge City, Kansas (**a**).

**Figure 7:**
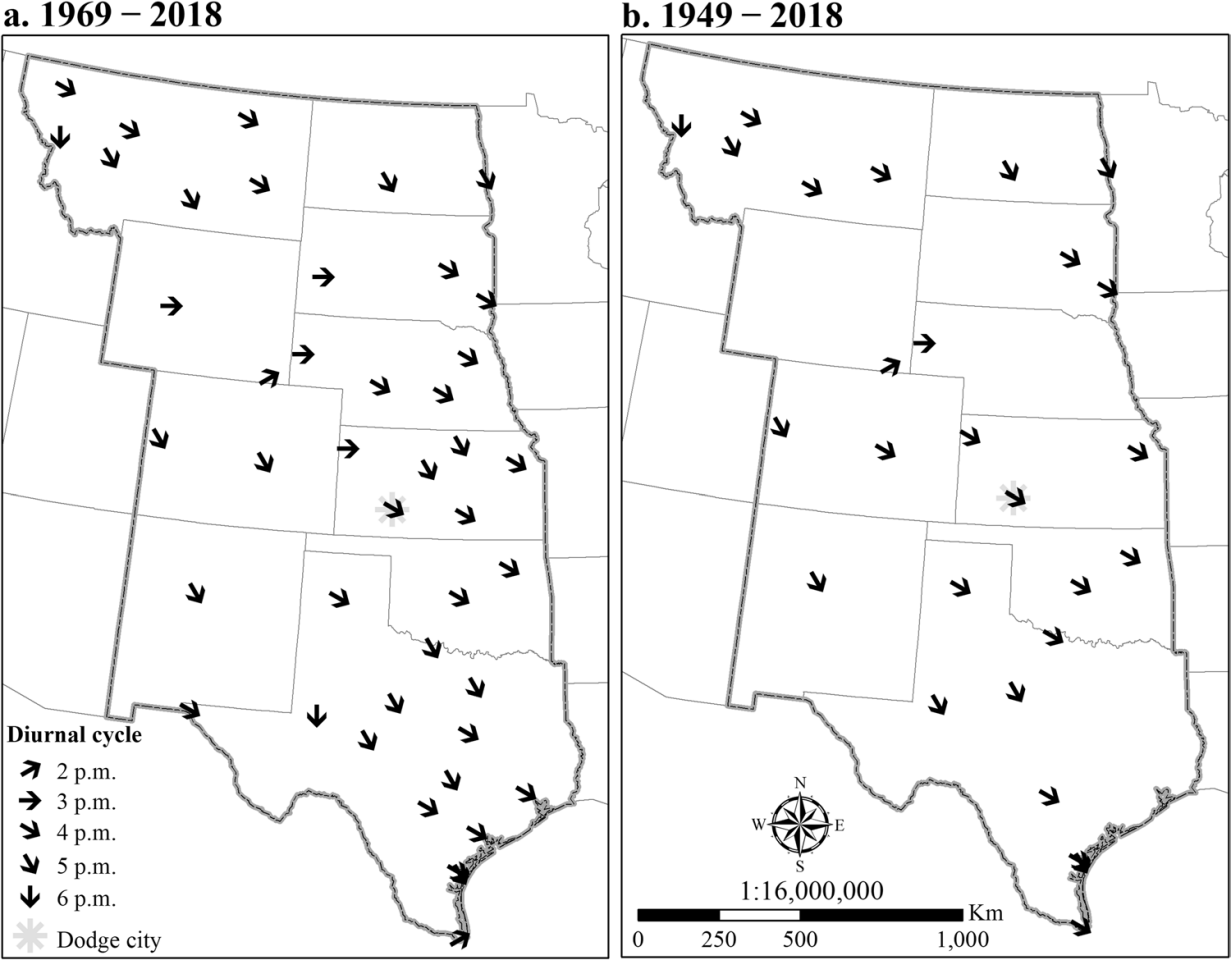
The highest diurnal frequency of HDWs in the stations for 50-years (**a**) and 70-years (**b**) periods. The figure has been generated with ESRI-ArcGIS, version 10.6 (https://www.esri.com/arcgis/about-arcgis).

An afternoon maximum is a predominant characteristic of the diurnal pattern of HDW events (Fig. 6). Hourly observations document that HDWs occur between 10 a.m. and 11 p.m. with a maximum frequency in the afternoon (4:00 p.m. and 5:00 p.m. local time). However, The highest diurnal frequency of HDWs was different in diverse stations ranging between 2:00 p.m. and 6:00 p.m. (Fig. 7).

The statistical *t* test analysis showed no statistically significant difference on the diurnal pattern of HDWs between 50-year and 70-year periods. Analyzing the separate components of HDWs, extreme high temperature events were most limiting for the occurrence of HDW events (Fig. 6). Same as HDWs, the maximum occurrence of extreme high temperature events, extreme low relative humidity, and extreme high wind speed were all discovered at 4:00 and 5:00 p.m. Hourly analysis of the three components documents that wind and RH can meet HDW criteria at any hour of the day, but temperature exceedance is limited to 10 a.m. to 11 p.m. (Fig. 6b,c).

**Figure 8:**
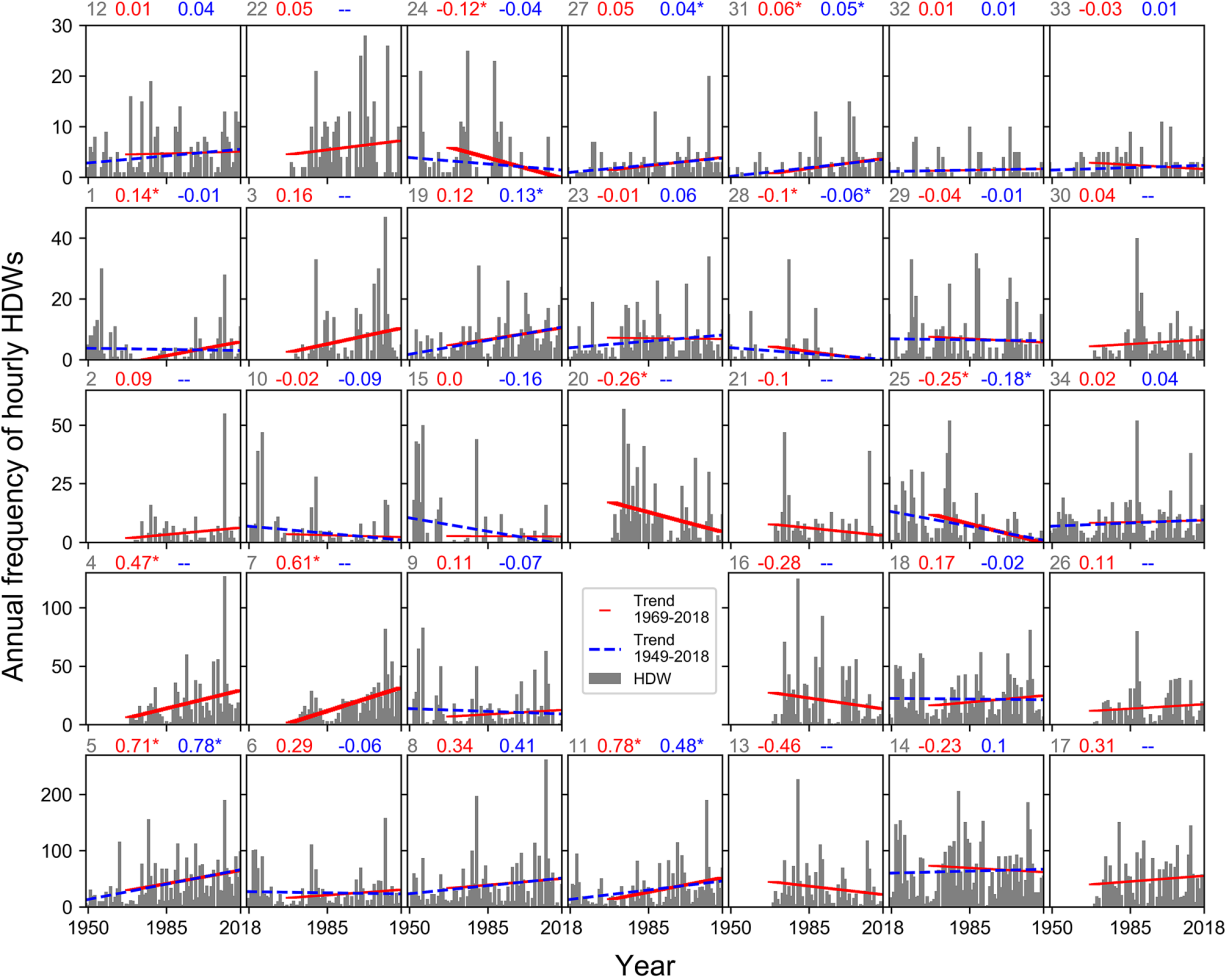
The annual frequency of hourly HDWs at stations that experience at least one HDW in each warm season. Each plot shows results for one station. The geographical location of stations is indicated by the first number on the top of each plot and the associated number locations shown in Fig. 9. Plots are sorted based on the maximum number of HDWs. The dashed blue line shows the trend for HDWs from 1949 to 2018 and the solid red line shows the trend from 1969 to 2018. Plots with no blue line do not include data for the 70-years period. The slope of the trend for 1949–2018 (blue) and 1969–2018 (red) are indicated on top of the plots. Significant trends are specified with a star next to the trend slope.

The Mann–Kendall trend test was used to analyze any station-based trend in the frequency of HDWs. From 1949–2018 and using annual totals, 30% (75% positive) of the stations showed a significant trend (Fig. 8). When analyzed on a monthly basis, August had the highest percentage of significant trends (18%; 11% positive). Negative trends in August occurred in eastern areas of South Dakota and North Dakota. Other months did not have stations with a decreasing trend. Positive monthly trends were most significant in Texas, Kansas, Colorado, and Montana.

**Figure 9:**
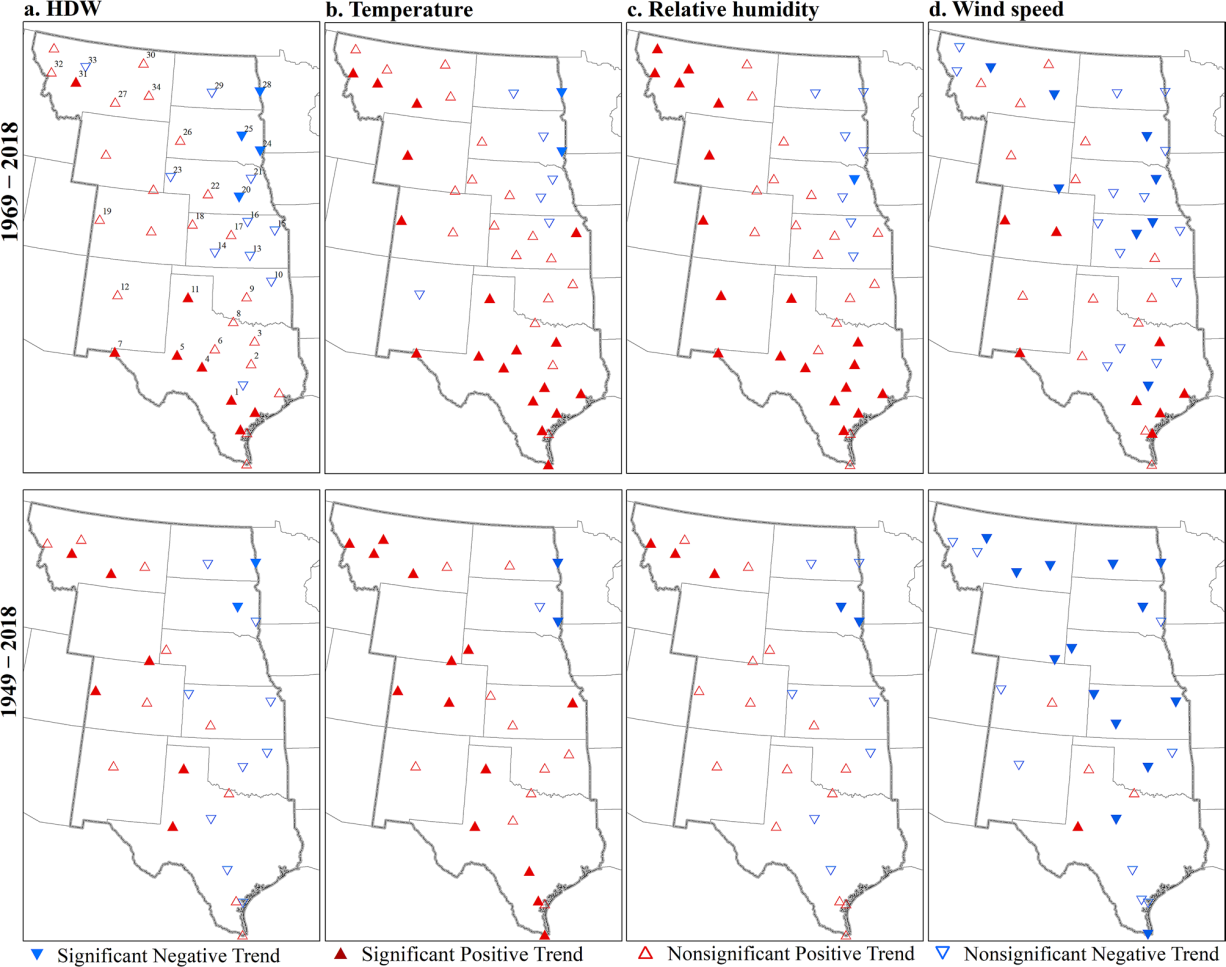
Trends of the frequency of HDWs (**a**), extreme high temperature (**b**), extreme low relative humidity (**c**), and extreme high wind speed (**d**) at each station in 50-years (top) and 70-years (bottom) periods. The figure has been generated with ESRI-ArcGIS, version 10.6 (https://www.esri.com/arcgis/about-arcgis).

From 1969–2018, 27% (67% positive) of all stations showed a significant trend in the annual HDW event total. For this analysis period, the majority of significant positive trends (87%) were in Texas. This finding can be linked to the higher number of HDW events associated with dry periods later in the time series (e.g., 2011). All statistically significant negative trends occurred in eastern portions of Nebraska, South Dakota, and North Dakota. The decrease was consistent with the decrease of extreme low humidity, high temperature, and wind speed. Analysis of monthly frequencies (Table 2) documented that May had increases in the occurrence of HDWs (18% stations) with no downward trends. Trend analysis for the September frequency values showed no significant trend in the occurrence of HDWs in either time periods. Dodge City with the highest frequency of HDWs showed a nonsignificant positive trend over the 70-years period and a nonsignificant negative trend over the 50-years period. The different pattern of trends at stations over the periods 1949–2018 and 1969–2018 demonstrates the importance of data period on the results. It is interesting to note that when the data are summarized for the entire warm season (compared to individual monthly results), a larger number of stations have a statistically significant trend. Figure 8 shows the temporal changes of HDWs at stations that experience at least one HDW in each warm season. Station location is identified in Fig. 9.

**Table 2:** Two-tailed Mann–Kendall trend test results indicate the temporal changes of HDW events in the central United States.

Time	1969–2018			1949–2018		
	Significant	Positive	Negative	Significant	Positive	Negative
Warm season	27.3	18.2	9.1	29.6	22.2	7.4
May	18.2	18.2	0.0	11.1	11.1	0.0
June	9.1	9.1	0.0	7.4	7.4	0.0
July	11.4	4.5	6.8	11.1	11.1	0.0
August	11.4	9.1	2.3	18.5	11.1	7.4
September	0.0	0.0	0.0	0.0	0.0	0.0

The values indicate the percentage of stations with a significant positive or negative trend with α = 0.05 in 50-years (1969–2018) and 70-years (1949–2018) periods.

Trends in extremes of temperature, wind speed, and relative humidity were also analyzed to understand the influence of each variable on temporal changes in HWDs (Fig. 9). For the 50-years period, 45% of stations had a significant trend for extreme temperature (41% positive) and relative humidity (43% positive) events. Among stations that had a significant trend for high wind speed in the 50-years period (36%), there was a similar number of stations with positive and negative trends (18%). For the 70-years period, 59% of stations had a significant trend in extreme temperature events that were mostly positive (52%). The changes in extreme low relative humidity were less significant (19%; 11% positive) over the longer period. However, high wind speed had a significant negative trend for a majority (52%) of stations. Only one station located in Texas had a significant positive trend for high wind speed in the 70-years period (Fig. 9).

The changes in extreme temperature events are consistent with global climate change^2,29,30^. Only two stations located in the north-eastern part of the study area had a significant negative trend for both time periods. This area might be considered as part of the “warming hole” in the United States where temperature and extreme temperature events have downward trends^68–72^. Future changes in the occurrence of compound extreme hot and dry days during crop-growing seasons will negatively influence crop yield^73^.

Although water vapor is increasing in the atmosphere^2,70,74^, the frequency of extreme low humidity events has also been increasing in western Great Plains. At 57% of the stations, the upward trend in extreme temperature events corresponds with an upward trend in the frequency of low relative humidity events in the 50-years period. Applying coupled global climate models, an increase was discovered in the annual frequency of dry days in the central United States^75^. When the time series does not include the southern Great Plains drought of the 1950s, the upward trends in extreme temperature and low relative humidity are statistically significant for stations across Texas. The downward trend of extreme wind events is consistent with previous study^36^ in which a decline was discovered in the 90th percentile and annual mean wind speed in the United States. Here, a decrease in HDWs was discovered at the stations with a decline in all three single extremes (Fig. 9). However, an increase of HDWs may affect water resource management challenges associated with the greater evapotranspiration^18^.

## Summary and conclusions

Spatial and temporal variations of HDWs were analyzed for the central United States (including the Great Plains) over two different periods of 1969–2018 (50-years) and 1949–2018 (70-years). For the 50-years period, there were more stations (44 stations) compared to the 70-years period (27 stations) that helped lead to a better understanding of spatial patterns. HDWs were defined as compound hourly events with high wind speeds (higher than or equal to 7 m/s), high temperature (higher than 35 °C), and low relative humidity (lower than 30%). Frequency analysis showed a greater occurrence of HDWs in western Kansas southward into Texas. This was consistent with the spatial pattern of extreme wind (wind speeds higher than 7 m/s) and extreme temperature (temperatures higher than 35 °C) events. The monthly analysis showed a greater probability of HDWs in July, with fewer occurrences in May and September. Compound HDW events were mainly observed in the afternoon with the highest frequencies at 4:00 and 5:00 p.m.

Results document the influence of the data period on the trend analysis. However, most stations located in Texas and the western Great Plains showed an upward trend in the HDWs. A downward trend in HDWs was found in the northeastern portion of the study region, consistent with the decrease in the extreme temperature, humidity, and wind speed events over time. Temporally, the central United States experienced large numbers of HDWs in 1980 and 2011. Two major, concurrent drought and heat waves in the summers of 1980 and 2011 coincided with the higher frequency of HDWs.

The higher temperatures associated with climate change may increase the frequency of extreme HDWs. HDW events have implications not only for those involved in crop production. Wind-driven wildfires and increased evapotranspiration effects on water management systems are potential impacts of these compound extreme meteorological events. Adaptation and mitigation strategies may need to be adjusted to cope with the negative impacts of these events.

## Methods

The HDW definition advanced by Leathers and Harrington^9^ was used in this study. After calculating the frequency of HDWs in each annual warm season, changes in the frequency of these events were analyzed spatially and temporally, considering frequency in different years and individual months. Then, the Mann–Kendall trend test^76,77^, which is widely used for climate variables^78–80^, was applied to determine the existence of any monotonic trend in the frequency of HDWs over time. In addition, the widely used Pearson and Spearman correlation test^81,82^ was applied to look for associations between HDWs and extreme temperature, humidity, and wind speed. A two-sided significant level of 0.05 was used for all parameters. For HadISd data, all times are provided as coordinated universal time (UTC). However, the central United States contains two different time zones, Mountain and Central. To better understand the diurnal changes of HDWs, the stations were separated based on their time zone and then local time was calculated for each station. Average linkage^60^ from hierarchical clustering methods and K-mean^61,62^ from non-hierarchical or partitional clustering methods were selected to cluster stations using R packages^83,84^.

### Study area

Previous studies^9,11^ showed the greatest probability of HDWs in the Great Plains in the central United States. The higher number of extreme HDWs in this region can be explained in part by the relatively high mean annual wind speed and a summer maximum in mean wind speeds^37^. The relative flatness of the Great Plains and a lack of tree cover are contributing factors. With an area of 2,898,107 km^2^, the ten states that contain the Great Plains span from Texas in the south to Canada in the north, and from the Rocky Mountains eastward to Kansas. In this study, weather observing sites within 10 states of Montana, Wyoming, Colorado, New Mexico, North Dakota, South Dakota, Nebraska, Kansas, Oklahoma, and Texas, were analyzed.

### Data

Sub-daily temperatures, relative humidity, and wind speed data were obtained from HadISD for 1949–2018 to analyze the long-term (70-years) changes of compound HDWs in the central United States. HadISD (version 3.0.1.201906p) is a station-based, sub-daily, quality-controlled dataset available from the Met Office website (https://www.metoffice.gov.uk/hadobs/hadisd). The dataset uses a subset of the Integrated Surface Database (ISD)^85^ from the National Oceanic and Atmospheric Administration (NOAA) National Centre for Environmental Information (NCEI), which initially provided data with temporal coverage beginning in 1973^86^. The HadISD provides the NCEI data in a more easily accessible format. They also have performed more detailed quality control on data and merged some stations to make longer records where this was reasonable to do so^86,87^. An update of the HadISD data added new stations and extended the temporal coverage back to 1931^87^. The most recent version of HadISD data includes 7677 stations with global coverage filtered by updated quality-control methods^87^. The new sets of HadISD data contain sub-daily humidity and heat-health measurements (e.g., heat index and apparent temperature). Stations with less than 10% missing data were selected for this study. In addition, stations with an entire year missing were excluded from the study to prevent bias in the results of temporal trend analysis.
